# Assessment of inflammation levels through the modified systemic inflammation score in patients with nonneovascular and neovascular age-related macular degeneration

**DOI:** 10.1097/MD.0000000000043047

**Published:** 2025-06-27

**Authors:** Burcu Polat Gültekin, Sebile Çomçali, Elif Şahin

**Affiliations:** a Department of Ophthalmology, Izmir City Hospital, Izmir, Turkey; b Department of Ophthalmology, Bilkent City Hospital, Ankara, Turkey.

**Keywords:** age-related macular degeneration, inflammation, modified systemic inflammation score

## Abstract

This study assesses the inflammation levels through the modified systemic inflammation score (mSIS) in patients with nonneovascular and neovascular age-related macular degeneration (AMD). A total of 90 participants were categorized into 3 groups: a control group, individuals with nonneovascular AMD under follow-up, and individuals with neovascular AMD who had not yet started injection treatment. Demographic and clinical parameters, including body mass index, were analyzed. The mSIS, based on serum albumin levels and the lymphocyte-to-monocyte ratio, was used to determine the level of systemic inflammation. There were no significant differences between the groups in terms of age (*P* = .08) or gender (*P* = .06). Regarding body mass index, no significant differences were observed between the groups (*P* = .06). When comparing mSIS scores between the groups, no significant differences were found for mSIS scores 0 and 1 across the groups. However, for mSIS 2, the proportion of participants with an mSIS score of 2 was significantly higher in the neovascular AMD group compared with the control group (*P* = .01). These findings suggest that mSIS may be a valuable tool for assessing systemic inflammation in AMD. The higher mSIS scores in the neovascular AMD group may indicate that disease severity is associated with increased inflammation. Further large-scale studies are needed to confirm the utility and clinical relevance.

## 1. Introduction

Age-related macular degeneration (AMD) is a major cause of irreversible blindness in older adults, posing a significant threat to central vision essential for critical tasks.^[1]^ While known risk factors include age, smoking, and genetics, recent evidence suggests that inflammation and oxidative stress play a significant role in AMD pathogenesis.^[2]^ The Beaver Dam Eye Study identified serum high-sensitivity C-reactive protein, tumor necrosis factor-alpha receptor-2, and interleukin-6 levels associated with early-stage AMD.^[3]^

In the literature, the neutrophil-to-lymphocyte ratio (NLR) has been shown to increase in various ocular conditions, such as keratoconus, glaucoma, pterygium, and idiopathic epiretinal membrane, due to its link with the cellular immune response.^[4]^ The modified systemic inflammation score (mSIS), which incorporates serum albumin levels and the lymphocyte-to-monocyte ratio (LMR), is a systemic inflammation scoring index commonly used to evaluate prognosis in various cancer types.^[5–7]^ According to this scoring system, a score of 0 is assigned when albumin levels are ≥4.0 g/dL and LMR is ≥3.4. A score of 1 is given if either albumin levels are <4.0 g/dL or LMR is <3.4, while a score of 2 is assigned when both albumin levels are <4.0 g/dL and LMR < 3.4. Chronic inflammation suppresses albumin synthesis in the liver, leading to hypoalbuminemia. In addition, lymphocytes and monocytes, as key cellular components of the immune system, produce chemokines and cytokines that drive the immune response and contribute to systemic inflammation.^[8]^ Chang et al^[9]^ indicated that this scoring system may serve as an objective marker, reflecting the balance between host inflammation and immune response status, to predict postoperative prognosis in patients with clear-cell renal cell carcinoma.

Previous studies have explored inflammation in various ocular diseases using the NLR as a biomarker of inflammation, reporting that NLR correlates with disease severity in AMD cases.^[10,11]^ To the best of our knowledge, the association between mSIS scores and AMD in the context of inflammation has not been evaluated to date. Therefore, this study aimed to assess inflammation levels in patients with nonneovascular and neovascular AMD, as well as in control groups, using the mSIS and to explore its potential diagnostic value.

## 2. Methods

A total of 90 participants were enrolled in this prospective study and divided into 3 groups: a control group consisting of 30 individuals without AMD; 30 individuals with nonneovascular AMD under follow-up; and 30 individuals newly diagnosed with neovascular AMD who had not yet initiated injection treatment. Informed consent was obtained from all participants, and the study was approved by the institutional review board of Ankara Bilkent City Hospital. All procedures adhered to the principles outlined in the Declaration of Helsinki.

All participants underwent a thorough ophthalmological evaluation, including biomicroscopic examination of the anterior and posterior segments and intraocular pressure measurements. Inclusion criteria were individuals over 50 years of age, either under follow-up for AMD or visiting the ophthalmology clinic without AMD.

Nonneovascular AMD cases included early and intermediate phases, excluding atrophic AMD. Patients with AMD were examined through a dilated pupil using fundus photography, fundus autofluorescence, and spectral-domain optical coherence tomography (Cirrus; Zeiss, Carl Zeiss Meditec, Dublin). Patients who were imaged at initial presentation and had intraretinal/subretinal fluid and/or choroidal neovascular membrane detected on spectral-domain optical coherence tomography, confirmed by fluorescein angiography, were included in the neovascular AMD group. Systemic conditions such as diabetes, hypertension, and smoking status were documented. Exclusion criteria included a history of ocular surgery, retinopathy due to systemic diseases, glaucoma, or ocular conditions such as optic neuropathy. Additional exclusions applied to individuals with systemic inflammatory diseases, hematologic disorders, active infections, steroid use within the last 3 months, cancer, and hepatic or renal diseases.

Clinical data collected included demographic variables such as age, gender, and body mass index (BMI). Complete blood count and biochemical tests were performed in the controlled hospital laboratory following an 8-hour fasting period, with blood samples processed within 30 minutes of collection, routinely performed in clinical settings. LMR was calculated as the ratio of the total lymphocyte count to the total monocyte count. The mSIS score was calculated using the following criteria: a score of 0 was assigned for albumin levels ≥ 4.0 g/dL and LMR ≥ 3.4, a score of 1 for either albumin levels < 4.0 g/dL or LMR < 3.4, and a score of 2 for both albumin levels < 4.0 g/dL and LMR < 3.4.^[12]^ The scores were then evaluated statistically.

### 2.1. Statistical analysis

Demographic data were presented as mean, SD, and percentage values. Statistical analysis for sample size calculation was performed using G*Power software, with an *α* level of 0.05 for statistical significance and a power of 60%. The analysis was performed with at least 30 participants in each group.

The analysis of variance test was used for comparisons among the groups, while logistic regression analysis was performed to evaluate the relationship between variables and inflammation levels. A *P* value of <.05 was considered statistically significant. For posthoc comparisons, the Tukey honestly significant difference test was applied.

## 3. Results

In the control group, 12 (40%) participants were female and 18 (60%) were male, with a mean age of 66.9 ± 10.3 (range: 50–82) years. In the nonneovascular AMD group, 21 (70%) participants were female and 9 (30%) were male, with a mean age of 69.5 ± 7.6 (range: 53–83) years. Meanwhile, the neovascular AMD group consisted of 17 (56.7%) participants who were female and 13 (43.3%) who were male, with an average age of 72.1 ± 7.5 (range: 55–84) years. There were no significant differences between the groups in terms of age (*P* = .08) or gender (*P* = .06). Regarding BMI, the control group had a mean BMI of 26.57 ± 4.9 kg/m^2^, the nonneovascular AMD group had 28.25 ± 6.3 kg/m^2^, and the neovascular AMD group had 29.98 ± 4.9 kg/m^2^. No significant differences were observed between the groups (*P* = .06). Table 1 presents the demographic data of the participants.

**Table 1:** Comparison of demographics and clinical features among the study groups.

Characteristics	Control group	Nonneovascular AMD	Neovascular AMD	*P* value
Age (years ± SD)	66.9 ± 10.3	69.5 ± 7.6	72.1 ± 7.5	.08
Gender (female/male, %)	12/18 (40/60)	21/9 (70/30)	17/13 (56.7/43.3)	.06
BMI (kg/m^2^)	26.57 ± 4.9	28.25 ± 6.3	29.98 ± 4.9	.06
Lymphocyte (/µL)	2.20 ± 0.68	2.20 ± 0.53	2.02 ± 0.57	.40
Monocyte (/µL)	0.41 ± 0.12	0.57 ± 0.37	0.42 ± 0.11	.01*
LMR	5.58 ± 1.8	4.70 ± 2.11	4.92 ± 1.81	.18
Albumin (g/dL)	4.39 ± 0.36	4.27 ± 0.40	4.1 ± 0.48	.13

AMD = age-related macular degeneration, BMI = body mass index, LMR = lymphocyte-to-monocyte ratio.

* Analysis of variance test.

When evaluating lymphocyte and monocyte levels, lymphocyte levels were comparable across all groups (*P* = .40). However, monocyte levels were significantly lower in both the control and neovascular groups compared with the nonneovascular group (*P* = .01). The LMR ratio and albumin levels, which are components of mSIS, were found to be similar across all groups (*P* > .05).

The participants were categorized into 3 groups based on their mSIS scores. In the control group, 18 (60%) participants had an mSIS score of 0, 10 (33.3%) participants had a score of 1, and 2 (6.7%) participants had a score of 2. In the nonneovascular AMD group, 14 (46.7%) participants had an mSIS score of 0, 10 (33.3%) participants had a score of 1, and 6 (20%) participants had a score of 2. Similarly, in the neovascular AMD group, 12 (40%) participants had an mSIS score of 0, 9 (30%) participants had a score of 1, and another 9 (30%) participants had a score of 2. When comparing mSIS scores between the groups, scores of 0 and 1 were similar across the groups. In contrast, in the mSIS 2 group, the proportion of participants with an mSIS score of 2 was significantly higher in the neovascular AMD group compared with the control group (*P* = .03; Table 2).

**Table 2:** The ratio of mSIS score among the groups.

mSIS score	Control group	Nonneovascular AMD group	Neovascular AMD group	*P* value
mSIS 0, n (%)	18 (60)	14 (46.7)	12 (40)	.91
mSIS 1, n (%)	10 (33.3)	10 (33.3)	9 (30)	.97
mSIS 2, n (%)	2 (6.7)	6 (20)	9 (30)	.03*

AMD = age-related macular degeneration, mSIS = modified systemic inflammation score.

* Analysis of variance test with Tukey honestly significant difference posthoc analysis.

**Figure 1.**
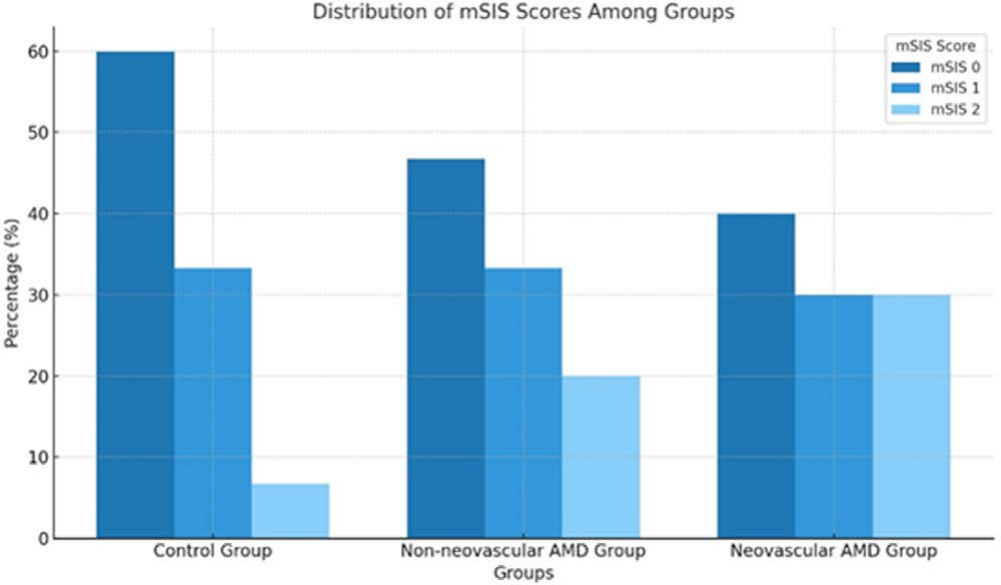
The distribution of mSIS scores among groups. AMD = age-related macular degeneration, mSIS = modified systemic inflammation score.

Post hoc analysis revealed that the proportion of participants with an mSIS score of 2 was similar between the nonneovascular and neovascular AMD groups (*P* = .40), as well as between the control group and the nonneovascular AMD group (*P* = .18). Notably, the neovascular AMD group exhibited significantly higher mSIS 2 rates compared with the control group (*P* = .01). The distribution of mSIS scores among the groups is depicted in Figure 1.

No significant relationship was observed between age, BMI, and smoking status and the mSIS score in the regression analysis (*P* > .05). In addition, no significant differences were found between the groups regarding smoking (4/4/5 cases, fewer than 10 cigarettes/d; *P* = .91), diabetes mellitus (5/5/6 cases; *P* = .93), or hypertension (3/4/6 cases; *P* = .53) in the control, nonneovascular AMD, and neovascular AMD groups, respectively.

## 4. Discussion

The exact etiology of AMD remains multifactorial, involving both genetic predispositions and environmental influences. Oxidative stress, inflammation, and abnormal lipid metabolism are major contributors to its pathophysiology.^[13]^ Chronic inflammation, in particular, accelerates damage to the retinal pigment epithelium and Bruch membrane, leading to drusen formation and subsequent macular dysfunction.^[14]^ Multiple immune components, including complement proteins, cytokines, and signaling pathways, are linked to AMD, suggesting various inflammatory routes may lead to the same disease.^[15]^

Several inflammatory markers, such as C-reactive protein, interleukin-6, and tumor necrosis factor-alpha, have been linked to AMD progression. These cytokines play a role in systemic inflammation, which subsequently impacts local inflammation within the eye.^[16]^ Considering the critical role of inflammation in AMD pathogenesis, the mSIS, which incorporates albumin levels and LMR, holds potential as a prognostic inflammatory biomarker for systemic inflammation in patients with AMD. Furthermore, the previously reported association between elevated white blood cell counts and early AMD, along with the observation of choroidal macrophages in cases with neovascular membranes, has strengthened the link between inflammation and AMD.^[17,18]^

The mSIS is an emerging tool for evaluating overall inflammatory status in various diseases, including cardiovascular conditions, cancers, and potentially, AMD.^[7,19,20]^ The evidence provided by Chang et al^[9]^ in renal cell carcinoma cases indicated that serum albumin levels and LMR are independent prognostic markers according to their multivariate analysis. These parameters were described as the mSIS and have been recognized as prognostic markers in various cancer types, highlighting the relationship between systemic inflammation and prognosis. Serum albumin, a protein produced by the liver, plays a critical role in maintaining osmotic pressure in the blood. Reduced albumin levels indicate a systemic inflammatory response and poor nutritional status. On the other hand, circulating monocytes, which differentiate into macrophages, are key contributors to vascularization progression. As a result, the mSIS, which integrates albumin levels and the LMR, serves as a diagnostic scoring system that reflects the immune response and systemic inflammation.^[21]^

In the present study, the proportion of participants with an mSIS score of 2 was significantly higher in the neovascular AMD group compared with the control group (*P* = .03). These findings suggest that the mSIS score, which reflects the level of systemic inflammation during the transition to neovascularization in AMD, could serve as a useful and noninvasive method for predicting inflammation status in AMD. In addition, patients with a lower mSIS, indicating reduced inflammation, may experience slower disease progression compared to those with higher scores. Moreover, the similar rates of mSIS 2 scores in both nonneovascular and neovascular AMD groups highlight the presence of a chronic inflammatory process in both forms of the disease.

Given the chronic nature of AMD and its potential to cause vision loss, early identification of patients at higher risk of disease progression is crucial. The mSIS, being a simple, cost-effective, and easily measurable tool, could be integrated into routine clinical practice to stratify patients based on their inflammatory status. Patients with higher mSIS scores may require closer monitoring to prevent irreversible vision loss.

The limitations of our study include a relatively small sample size. In addition, the heterogeneity of AMD, influenced by factors such as diet and genetic predispositions, could potentially affect the scores. Furthermore, early and intermediate AMD cases comprised the nonneovascular group, as atrophic AMD may involve distinct pathways contributing to retinal cell death.

The mSIS is a promising tool for evaluating systemic inflammation in AMD. Elevated mSIS levels, particularly in the neovascular form of AMD, highlight the critical role of inflammation and may serve as a biomarker for clinicians to assess the risk of neovascularization. On the basis of the results of our study, we believe it is possible to assess the relationship between AMD and systemic inflammation from a different perspective.

## 5. Conclusion

Our findings suggest that systemic inflammation, as measured by mSIS, is more pronounced in neovascular AMD. These results highlight the potential role of systemic factors in the progression of the disease.

## Author contributions

**Conceptualization:** Burcu Polat Gültekin.

**Methodology:** Burcu Polat Gültekin, Sebile Çomçali, Elif Şahin.

**Supervision:** Burcu Polat Gültekin.

**Writing – original draft:** Burcu Polat Gültekin.

**Data curation:** Sebile Çomçali, Elif Şahin.

**Writing – review & editing:** Sebile Çomçali, Elif Şahin.
